# Humoral and Cellular Response of Frontline Health Care Workers Infected by SARS-CoV-2 in Nice, France: A Prospective Single-Center Cohort Study

**DOI:** 10.3389/fmed.2020.608804

**Published:** 2021-01-27

**Authors:** Marion Cremoni, Caroline Ruetsch, Kévin Zorzi, Céline Fernandez, Sonia Boyer-Suavet, Sylvia Benzaken, Elisa Demonchy, Jean Dellamonica, Carole Ichai, Vincent Esnault, Vesna Brglez, Barbara Seitz-Polski

**Affiliations:** ^1^Service de Néphrologie-Dialyse-Transplantation, CHU de Nice, Université Côte d'Azur, Nice, France; ^2^Unité de Recherche Clinique de la Côte d'Azur (UR2CA), Université Côte d'Azur, Nice, France; ^3^Laboratoire d'Immunologie, CHU de Nice, Université Côte d'Azur, Nice, France; ^4^Centre de Référence Maladies Rares Syndrome Néphrotique Idiopathique, CHU de Nice, Université Côte d'Azur, Nice, France; ^5^Service d'Infectiologie, CHU de Nice, Université Côte d'Azur, Nice, France; ^6^Service de Réanimation Médicale, CHU de Nice, Université Côte d'Azur, Nice, France; ^7^Service de Réanimation Médicochirurgicale, CHU de Nice, Université Côte d'Azur, Nice, France

**Keywords:** health care workers, SARS-CoV-2, COVID-19, humoral response, cellular response, blood immune biomarkers

## Abstract

Frontline health care workers (HCWs) have been particularly exposed to Severe Acute Respiratory Syndrome Coronavirus 2 (SARS-CoV-2) since the start of the pandemic but the clinical features and immune responses of those infected with SARS-CoV-2 have not been well described. In a prospective single center cohort study, we enrolled 196 frontline HCWs exposed to the SARS-Cov-2 and 60 patients with moderate and severe forms of the coronavirus disease 2019 (COVID-19). Serological tests and cytokines assay were performed to analyze SARS-CoV-2-specific humoral and cellular immunity. Of the 196 HCWs tested, 15% had specific antibodies against SARS-CoV-2 and 45% of seropositive HCWs were strictly asymptomatic. However, in comparison to moderate and severe forms, HCWs with mild or asymptomatic forms of COVID-19 showed lower specific IgA and IgG peaks, consistent with their mild symptoms, and a robust immune cellular response, illustrated by a high production of type I and II interferons. Further studies are needed to evaluate whether this interferon functional immune assay, routinely applicable, can be useful in predicting the risk of severe forms of COVID-19.

## Introduction

Following the first descriptions of acute respiratory syndrome cases in Wuhan, Hubei province, China, at the end of December 2019, a novel beta coronavirus called Severe Acute Respiratory Syndrome Coronavirus 2 (SARS-CoV-2) was identified (1). This virus, responsible for the new coronavirus disease (COVID-19), quickly spread to other regions of China and then outside the country. The pandemic stage was declared by the World Health Organization (WHO) on March 11, 2020.

The transmission of COVID-19 to health care workers (HCWs) is a serious concern as it puts potentially very vulnerable patient populations at risk. Nasopharyngeal swabs (NPSs) are being widely used as specimens for real-time reverse transcription (RT)-PCR to detect symptomatic HCWs (fever, cough, fatigue, muscle pain, diarrhea). This common practice helps to slow or stop the spread of infection and protect patients and other HCWs. However, a significant proportion of those infected were asymptomatic or pauci-symptomatic but still transmitted the virus (2–4).

As shown in previous studies, patients infected with SARS-CoV-2 develop an antibody response against the virus (5). Asymptomatic individuals, however, appear to reveal a weaker humoral immune response (6). Other studies, conducted in patients with moderate to severe forms of COVID-19, looked at the cellular immune response. They showed that lymphopenia (1, 7), and type I and II interferon (IFN) deficiency secreted by the remaining T cells (8–10) correlate with the severity of the disease. At present, this cellular immune response has not yet been studied in asymptomatic subjects.

To our knowledge, only few studies have been conducted characterizing both humoral and cellular immune response to SARS-CoV-2 infection (11), and no study investigated this global immune response in a specific population of frontline HCWs particularly exposed to SARS-CoV-2 during the pandemic. In this prospective single-centered cohort study, we first sought to assess the SARS-CoV-2 antibodies seroprevalence of asymptomatic and pauci-symptomatic SARS-CoV-2 infection in frontline health care workers, as well as compare their humoral and cellular response to patients with moderate and severe forms of COVID-19. In addition to improving knowledge on the immune response to this emerging disease, the identification of potential blood immune biomarkers predictive of the response to SARS-CoV-2 could allow us to better prevent the onset of severe forms of COVID-19, particularly in subjects highly exposed to the virus such as frontline HCWs.

## Materials and Methods

### Study Design and Participants

We performed a prospective cohort study of subjects exposed to SARS-CoV-2 virus at Nice University Hospital, France. For this, we included volunteer frontline health care workers (HCWs) defined as those working in units providing care for patients with confirmed COVID-19, in Nice University Hospital from April 15 to May 26, 2020. After signing an informed consent, they completed a self-questionnaire and had their blood drawn to perform a serological test and a functional immune assay. Exclusion criteria were: (1) pregnancy or breastfeeding; (2) HCWs having received previous immunosuppressive therapy for COVID-19 treatment. We divided seropositive SARS-CoV-2 HCWs into four subgroups according to the symptoms that occurred in the 3 months preceding the blood test and that they had to declare in the questionnaire: (1) strictly asymptomatic; (2) mild symptoms if they had common symptoms of COVID-19, including fever, fatigue, cough, rhinorrhea, muscle pain, headache, diarrhea, anosmia or other flu-like symptoms (1, 7); (3) moderate form of COVID-19 if they were hospitalized in infectious diseases units due to clinical symptoms associated with dyspnea and radiologic findings consistent with a COVID-19 pneumonia on thoracic CT-scan; (4) severe form of COVID-19 if they were either hospitalized or transferred to the intensive care unit with respiratory failure requiring mechanical ventilation, or with multiple organ failure. Household members of the HCWs tested seropositive for SARS-CoV-2 infection were also invited to participate in the study.

We performed a second prospective cohort study made up of patients infected with SARS-CoV-2 followed at Nice University Hospital, France. The inclusion criteria were: (1) all adult patients hospitalized for COVID-19 in infectious diseases units (IDU) or in intensive care unit (ICU), in Nice University Hospital from March 13 to April 16, 2020; (2) ability to sign an informed consent. Exclusion criteria were: (1) age under 18; (2) patients under custody, in prison or with a mental illness; (3) pregnancy or breastfeeding; (4) patients having received previous immunosuppressive therapy for COVID-19 treatment. The patients were divided into two groups according to the severity of infection with SARS-CoV-2: moderate or severe forms of COVID-19 as above. All patients presented a COVID-19 symptomatology according to WHO recommendations (12) with a CT-scan characteristic of COVID-19 (13) or two consecutive positive RT-PCR tests for SARS-CoV-2 on upper and lower respiratory tract specimens (NPS or invasive respiratory sample).

### Procedures

#### Data Collection

Epidemiological and clinical data were collected using the electronic medical records applications Clinicom® and ORBIS® for COVID-19 patients and the self-questionnaire for HCWs. This self-administered questionnaire collected information on demographic factors, medical history, previous or present treatments, hospital function, known risk factors for COVID-19, and symptoms that may have occurred in the 3 months preceding the blood sample. HCWs were also asked if they had already been tested for COVID-19 RT-PCR and what were the results. When available, the time delays (in days) between the onset of the first symptoms of COVID-19 and inclusion, i.e., the day of the first blood sampling, were recording. For asymptomatic IgA-positive HCWs without anti-SARS-CoV-2 IgG antibodies, we estimated this time to be between 7 and 10 days. We considered this data to be missing for asymptomatic HCWs who were IgG-positive with or without IgA antibodies.

#### Sampling Process

SARS-CoV-2 virological tests for patients followed the World Health Organization recommendations (12). NPSs were obtained by nurses or physicians using a standard technique and were immediately placed in a transport medium and delivered to our central laboratory to confirm COVID-19 by real-time reverse transcription-polymerase chain reaction (RT-PCR) methods. Blood samples were collected at day 0 of the admission and at several follow-up points up to 2 months after hospital admission for COVID-19 patients, and at inclusion for HCWs. For hospital staff tested positive for SARS-CoV-2 infection, a second blood sample was taken 1 month after inclusion. Samples were immediately processed and then frozen and stored at −20°C until serological tests and functional immune assay (cellular response/cytokines assay) were performed. Freeze-thaw cycles were minimized to preserve the quality of the samples.

### Laboratory Methods

#### Serological Test

Serological tests for anti-SARS-CoV-2 IgA and IgG isotypes antibodies were performed on serum using a commercially available enzyme-linked immunosorbent assays (ELISA) which used the S1-domain of the spike protein of SARS-CoV-2 as the antigen (Euroimmun AG, Lübeck, Germany, # EI 2606-9601 A and # EI 2606-9601 G). They were run on IF Sprinter IFT/ELISA (Euroimmun) according to the manufacturer's protocol. The results are evaluated by calculating the ratio between the optical density (OD) of the sample at 450 nm and the OD of the calibrator at 450 nm, according to the following formula:

$$\begin{array}{l} \frac{OD\;of\;the\;sample}{OD\;of\;the\;calibrator}=OD\;ratio \end{array}$$

According to the manufacturer's recommendations, the results were then interpreted as follows: OD ratio <0.8 = negative; ≥0.8 and <1.1 = indeterminate; ≥1.1 = positive (14).

#### Cellular Response/Cytokines Assay

One milliliter of whole blood was stimulated with immune ligands [anti-CD3 as T-cells stimulant, and R848 as Toll-like receptors 7/8 (TLR 7/8) agonist] on single lyophilized spheres (LyoSphere™, Qiagen) within 8 h from blood collection. Stimulated blood samples were incubated for 16–24 h at 37°C and then centrifuged at 2,000–3,000 × g for 15 min to harvest the stimulated supernatant. Levels of cytokines after non-specific stimulation were measured using IFN-γ ELISA microplates from QuantiFERON-Monitor test (Qiagen®) and Ella (ProteinSimple®) custom-designed cartridges for the detection of IFN-α, following the manufacturers' instructions.

### Statistical Analyses

For descriptive statistics, data are presented as mean and standard deviation for quantitative variables with Gaussian distribution, as median and range for quantitative variables with non-Gaussian distribution, or as numbers and percentages for qualitative variables. The Shapiro-Wilk test was used to determine if a variable had a Gaussian distribution or not. Quantitative variables were compared by the unpaired *t*-test or one-way ANOVA if the values were normally distributed and by the Mann-Whitney test if they were not. Qualitative variables were compared using Chi-square test or Fisher's exact test as appropriate. A Wilcoxon matched pairs signed rank test was used to compare two measurements of a quantitative variable. Statistical analyses were performed using GraphPad Prism 7.0 (GraphPad Software, Inc., San Diego, CA). Differences were considered significant when *P* value < 0.05.

### Ethics and Consent

The study protocol conformed to the ethical guidelines of the 1975 Declaration of Helsinki and was reviewed and approved by our local institutional review committee (NCT04355351). Written informed consent was obtained from participants prior to inclusion in the study. All collected data and samples were securely stored.

## Results

### Participants' Characteristics

**Figure 1:**
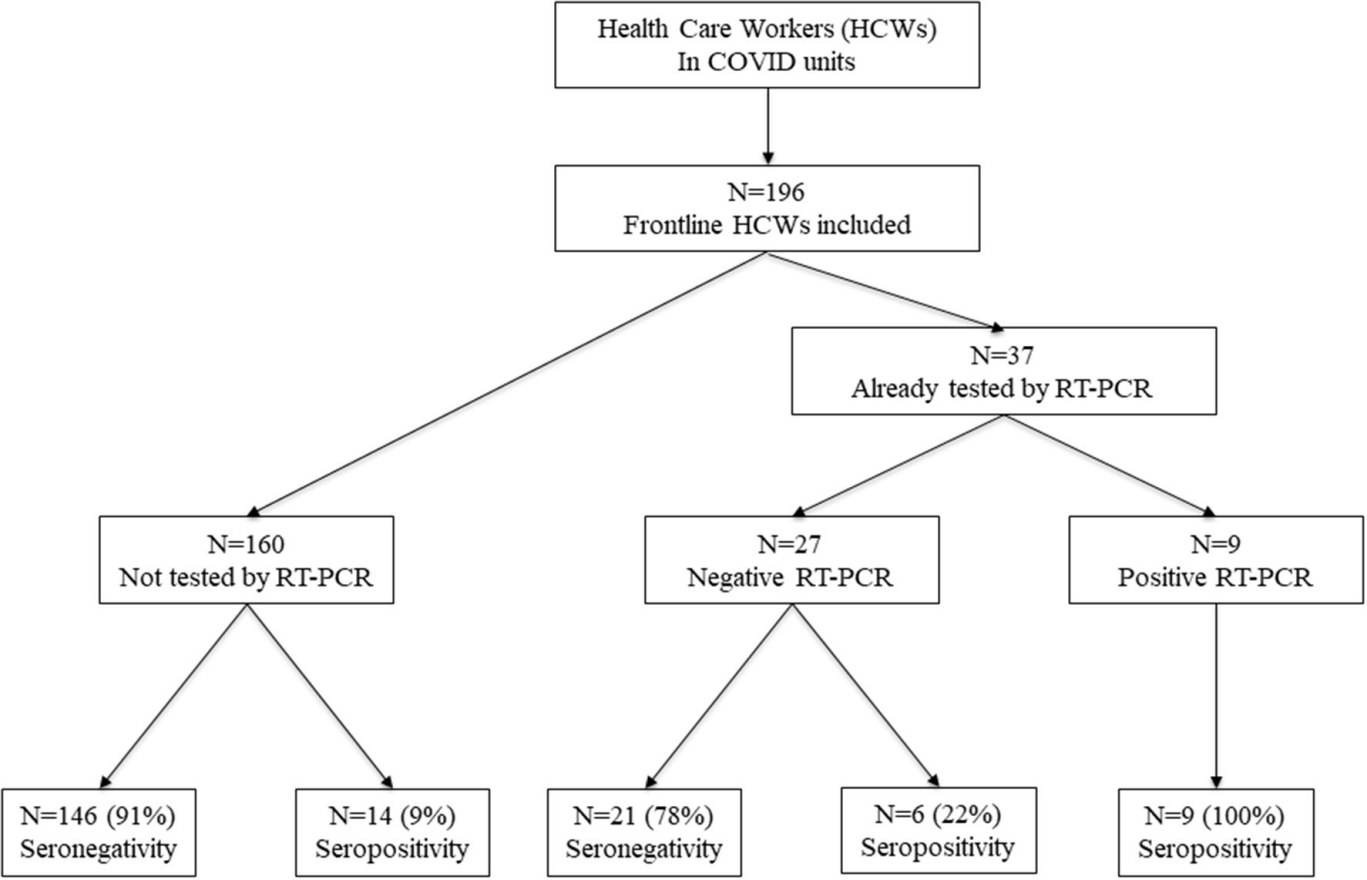
Enrollment of HCWs and the subgroups formed according to SARS-CoV-2 infection. HCWs, health care workers.

**Figure 2:**
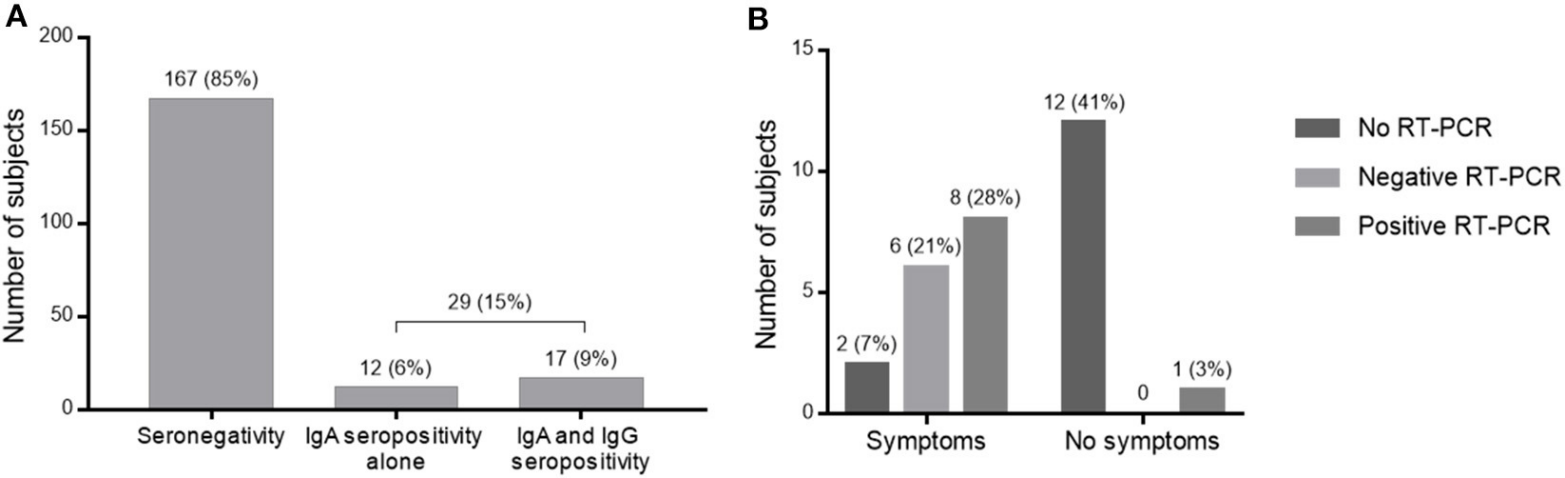
Results of serological tests. **(A)** Serological screening test for SARS-CoV-2 among HCWs in COVID units. **(B)** RT-PCR results and symptomatology of HCWs seropositive for SARS-CoV-2.

Between April 15 and May 26, 2020, we enrolled 196 frontline HCWs in Nice University Hospital. Twenty-nine (15%) were seropositive for anti-SARS-CoV-2 antibodies. Nine HCWs had a positive NPS: one with moderate symptoms of COVID-19 requiring hospitalization in infectious diseases unit (IDU), seven with mild symptoms and one asymptomatic subject but with close contact with a confirmed COVID-19 case. Twenty HCWs had no NPS but were found seropositive: eight had presented mild symptoms compatible with a COVID-19, and 12 were asymptomatic (Figure 1). Overall, 1/29 (3%) seropositive HCWs had moderate symptoms, 15/29 (52%) had mild symptoms of COVID-19, and 13/29 (45%) were strictly asymptomatic (Figure 2B).

Of these 29 infected HCWs, 12 presented only IgA antibodies and 17 had IgA and IgG seroconversion (Figure 2A). Among them, the nine infected HCWs who had a positive PCR had both IgA and IgG. The presence of IgA antibodies would indicate contamination more than 10 days ago with a sensitivity of 100% (14), while IgG detection would signify contamination more than 21 days ago with a sensitivity of 100% (14).

**Figure 3:**
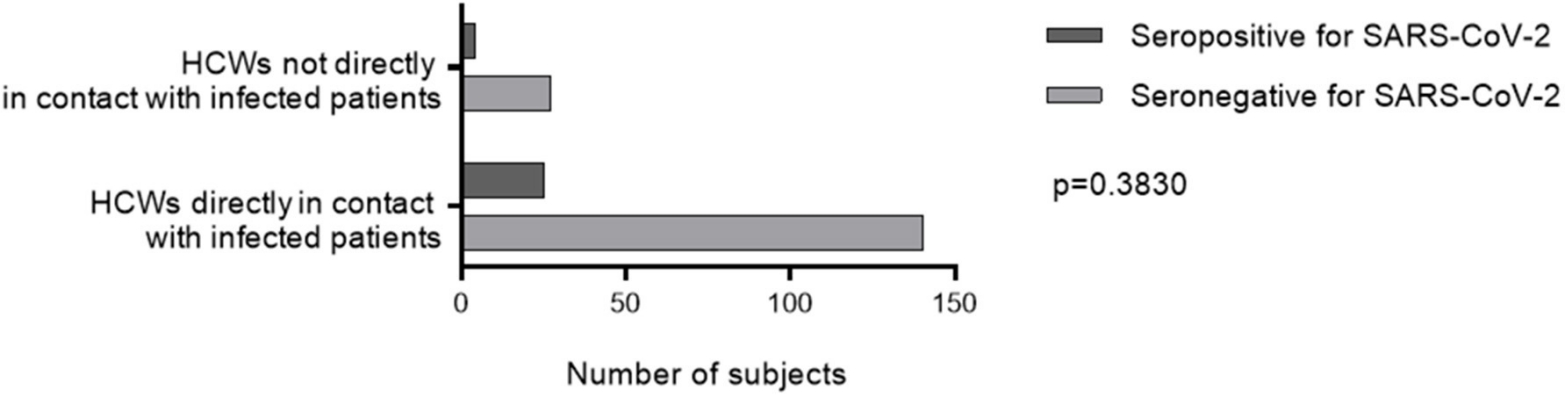
SARS-CoV-2 seropositivity rate according to occupational exposure. A two-way ANOVA test was used to compare the seropositivity rate HCWs directly exposed and those not directly exposed to infected patients. HCWs, health care workers.

Twenty-one (72%) infected HCWs were women with a median (IQR) age of 38 (31–43) years, while 23 (38%) infected patients were women with a median age of 65 (54–74) years, reflecting the high proportion of young women in health care. Most HCWs were nursing assistants [six seropositive for SARS-CoV-2 out of 32 tested (15%)], physicians [7/41 (17%)], nurses [8/53 (15%)] and medicine residents [4/32 (12.5%)] (Table 1). The other HCWs in the cohort were dietitians, nursing students, physiotherapists, and psychologists (none seropositive for SARS-CoV-2 out of seven tested). HCWs working in COVID units but not directly in contact with patients were hospital engineers [two seropositive for SARS-CoV-2 out of three tested (67%)], laboratory technicians [1/3 (33%)], hospital service agents [1/11 (9%)], senior health managers (0/9), medical secretaries (0/4) and clinical research assistants (0/1). We did not find any significant difference in the rate of SARS-CoV-2 infection between HCWs directly exposed and those not directly exposed to infected patients (*p* = 0.38) (Table 1, Figure 3).

**Table 1:** SARS-CoV-2 infection rate by function within the COVID unit.

**Function within the COVID unit**	**Seropositivity rate/total number of agents tested, *n*/*N* (%)**
**Hospital workers directly in contact with SARS-CoV-2 infected patients (*****n*** **=** **165)**	
Nursing assistants	6/32 (19%)
Physicians	7/41 (17%)
Nurses	8/53 (15%)
Medicine residents	4/32 (12.5%)
Dietitians	0/1 (0%)
Nursing students	0/3 (0%)
Physiotherapists	0/2 (0%)
Psychologists	0/1 (0%)
**Hospital workers not directly in contact with SARS-CoV-2 infected patients (*****n*** **=** **31)**	
Hospital engineers	2/3 (67%)
Laboratory technicians	1/3 (33%)
Hospital service agents	1/11 (9%)
Senior health managers	0/9 (0%)
Medical secretaries	0/4 (0%)
Clinical research assistants	0/1 (0%)

Between March 13 and April 16, 2020, we enrolled 60 patients with COVID-19 in Nice University Hospital, divided in two subgroups: moderate (*n* = 30) and severe cases of COVID-19 (*n* = 30). This cohort was compared to the frontline HCWs.

Demographic and baseline characteristics of the 29 HCWs and 60 patients with COVID-19, separated into three groups according to the severity of symptoms, are summarized in Tables 2, 3. The cohort of infected HCWs included significantly fewer men (8/29, 28%) than that of patients (37/60, 62%). The HCWs were also younger [38 (31–43) years] than the patients [65 (54–74) years]. The rate of comorbidities in affected HCWs was 31% (9/29), which is significantly lower than patients whose rate of comorbidities was 82% (49/60). The most common comorbidities among HCWs with SARS-CoV-2 infection were asthma [3 (10%)], hypertension [2 (7%)] and cancer [2 (7%)] while those among patients were hypertension [28 (47%)], diabetes [16 (27%)] and cardiovascular disease [10 (17%)]. There was no difference in taking treatments known to cause severe COVID-19 symptoms in the two cohorts. As in previous studies, being overweight defined by a BMI > 25 was found to be a risk factor for a severe form of COVID-19 (mean 22.76 in asymptomatic and mild cases, 25.31 in moderate cases and 27.02 in severe cases, global *p* value = 0.0005). In our study, there was the same rate of smokers in the three groups (*p* = 0.1941). Most of the infected HCWs were strictly asymptomatic [13 (45%)], but fever [9 (31%)], cough [7 (24%)], and headache [5 (17%)] were prevalent. In COVID-19 patients the three most common symptoms were dyspnea [44 (73%)], cough [38 (63%)], and fever [35 (58%)]. The median time from the onset of first symptoms of COVID-19 to inclusion, otherwise the date of first blood collection, was 7 (7–54) days for HCWs, 9 (5–14) days for patients with moderate COVID-19 infection and 8 (5–10) days for severe cases. There was no difference in demographics, comorbidities, and symptoms between HCWs in COVID-dedicated units who were directly in contact with infected patients, from HCWs not in direct contact with patients (data not shown).

**Table 2:** Baseline characteristics of health care workers and patients with COVID-19.

	**HCWs (*n* = 29)**	**Patients (*n* = 60)**	***P* value**
Age, years	38 (31–43)	65 (54–74)	<0.0001
Males, *n* (%)	8 (28%)	27 (45%)	0.1150
Any comorbidity, *n* (%)	9 (31%)	49 (82%)	<0.0001
Diabetes, *n* (%)	0 (0%)	16 (27%)	0.0021
Hypertension, *n* (%)	2 (7%)	28 (47%)	0.0002
Cardiovascular disease, *n* (%)	0 (0%)	10 (17%)	0.0196
COPD, *n* (%)	0 (0%)	2 (3%)	0.3200
Asthma, *n* (%)	3 (10%)	4 (7%)	0.5457
Cancer, *n* (%)	2 (7%)	8 (14%)	0.3675
**Previous treatment**			
NSAIDs, *n* (%)	0 (0%)	2 (3%)	0.3200
Corticosteroids, *n* (%)	1 (3%)	3 (5%)	0.7405
Immunosuppressive therapy, *n* (%)	1 (3%)	5 (8%)	0.3890
BMI	22.09 (20.33–23.78)	25.40 (23.06–29.19)	0.0003
Smoking, *n* (%)	3 (10%)	2 (3%)	0.1782

*BMI, body mass index; COPD, chronic obstructive pulmonary disease; HCWs, hospital care workers; NSAIDs, non-steroidal anti-inflammatory drugs*.

**Table 3:** Demographic and baseline characteristics of health care workers and patients with COVID-19 according to the severity of symptoms.

	**Asymptomatic and mild cases: HCWs after screening (*n* = 28)**	**Moderate cases: HCW, *n* = 1, and patients hospitalized in IDU, *n* = 30 (*n* = 31)**	**Severe cases: patients hospitalized in ICU (*n* = 30)**	**Global *P* value**
**Characteristics at baseline**				
Age, years	38 (31–43)	64 (54–75)	65 (53–72)	<0.0001
Males, *n* (%)	7 (25%)	17 (55%)	21 (70%)	0.0024
Any comorbidity, *n* (%)	9 (32%)	27 (87%)	22 (73%)	<0.0001
Diabetes, *n* (%)	0 (0%)	7 (23%)	9 (30%)	0.0086
Hypertension, *n* (%)	2 (7%)	15 (48%)	12 (40%)	0.0019
Cardiovascular disease, *n* (%)	0 (0%)	4 (13%)	6 (20%)	0.0513
COPD, *n* (%)	0 (0%)	2 (6%)	0 (0%)	0.1475
Asthma, *n* (%)	3 (11%)	2 (6%)	2 (7%)	0.7951
Cancer, *n* (%)	2 (7%)	3 (10%)	5 (17%)	0.4885
**Previous treatment**				
NSAIDs, *n* (%)	0 (0%)	1 (3%)	1 (3%)	0.6250
Corticosteroids, *n* (%)	1 (4%)	1 (3%)	2 (7%)	0.7782
Immunosuppressive therapy, *n* (%)	1 (4%)	2 (6%)	3 (10%)	0.6193
BMI	22.76 ± 4.33	25.31 ± 4.03	27.02 ± 5.17	0.0005
Smoking, *n* (%)	3 (11%)	0 (0%)	2 (7%)	0.1941
**Days after first signs of COVID-19**	7 (7–54)^a^	9 (5–14)^b^	8 (5–10)^c^	0.1363
**Signs and symptoms of COVID-19**				
Fever, *n* (%)	8 (29%)	20 (65%)	16 (53%)	0.0195
Cough, *n* (%)	6 (21%)	23 (74%)	16 (53%)	0.0003
Headache, *n* (%)	5 (18%)	5 (16%)	3 (10%)	0.6686
Muscle pain, *n* (%)	4 (14%)	8 (26%)	2 (7%)	0.1178
Dyspnea, *n* (%)	3 (11%)	22 (71%)	23 (77%)	<0.0001
Anosmia, *n* (%)	4 (14%)	5 (16%)	4 (13%)	0.9517
Diarrhea, *n* (%)	3 (11%)	9 (29%)	7 (23%)	0.2180

*BMI, body mass index; COPD, chronic obstructive pulmonary disease; HCWs, hospital care workers; ICU, intensive care unit; IDU, infectious disease unit; NSAIDs, non-steroidal anti-inflammatory drugs*.

a *data was missing for eight patients*,

b *data was missing for four patients*,

c *data was missing for four patients*.

### Humoral Immune Responses to SARS-CoV-2 in Health Care Workers and Patients

#### Kinetics of Specific Antibodies to SARS-CoV-2 in Severe COVID-19 Patients

**Figure 4:**
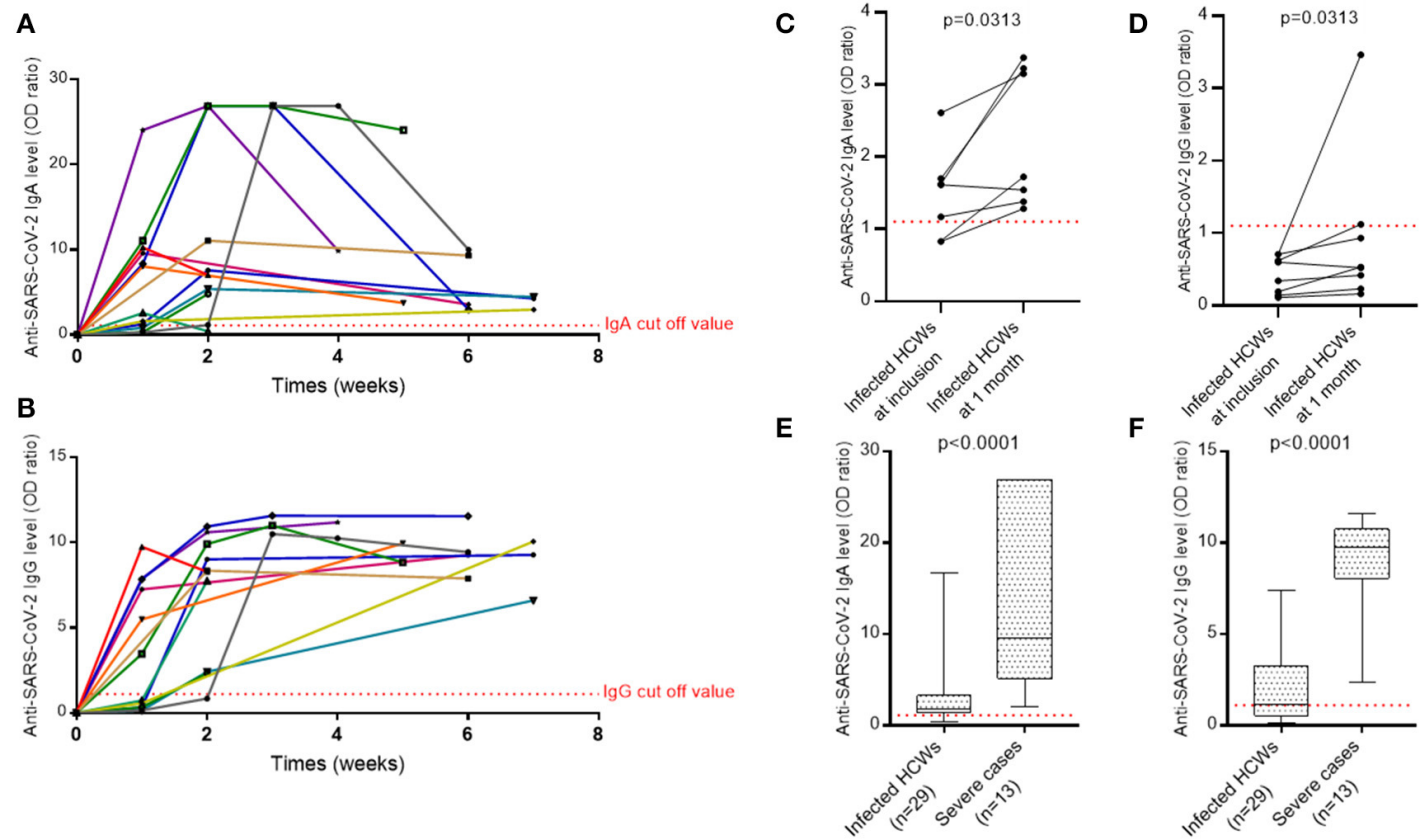
The evolution of antibody response against SARS-CoV-2. **(A)** IgA antibody response over time against the spike protein of SARS-CoV-2 in ICU patients. **(B)** IgG antibody response over time against the spike protein of SARS-CoV-2 in ICU patients. **(C)** IgA antibody response against the spike protein of SARS-CoV-2 in pauci- or asymptomatic HCWs. **(D)** IgG antibody response against the spike protein of SARS-CoV-2 in pauci- or asymptomatic HCWs. A Wilcoxon matched pairs signed rank test was used to compare anti-SARS-CoV-2 IgA and IgG levels at screening and at 1 month. **(E)** IgA levels of infected HCWs vs. severe cases. **(F)** IgG levels of infected HCWs vs. severe cases. A non-parametric two-tailed test (Mann-Whitney) was used to compare the IgA and IgG levels of infected HCWs to severe cases. Quantitative results of IgA and IgG levels were expressed in arbitrary units by OD ratio obtained by calculating the ratio of the OD of the sample over the OD of the calibrator (as described in Methods). Each colored line in **(A,B)** represents a patient. HCWs, health care workers.

We evaluated SARS-CoV-2 specific antibody responses in 13 severe cases who recovered from the infection using serum samples collected at day 0 of the admission and at several follow-up points up to 2 months after hospital admission. The proportion of patients with positive SARS-CoV-2-specific IgA and IgG at admission was 9/13 (69%) and 6/13 (46%), respectively, and reached 100% for the two isotypes after 15 days of hospitalization (Figures 4A,B). During the first 2 weeks after the admission for IgA and 4 weeks after the admission for IgG, titers for SARS-CoV-2-specific antibodies were generally increasing. The IgA level then decreased, although it was still positive even at 7 weeks, while that of IgG remained relatively stable over time.

#### Kinetics of Specific Antibodies to SARS-CoV-2 in Health Care Workers

For eight infected HCWs with only IgA at inclusion, a second serum sample was collected 1 month later to verify IgG seroconversion. The levels of IgA and IgG antibodies specific to SARS-CoV-2 increased significantly between the two time points but only two individuals achieved the level of IgG positivity and one exhibited an undetermined result (Figures 4C,D).

Levels of IgA and IgG in patients with severe COVID-19 were significantly higher than maximum levels obtained in infected HCWs [IgA: 9.59 (5.10–26.89) vs. 1.82 (1.37–3.29) respectively, *p* < 0.0001; IgG: 9.75 (8.05–10.75) vs. 1.12 (0.52–3.24) respectively, *p* < 0.0001] (Figures 4E,F).

#### Detection of Specific Antibodies to SARS-CoV-2 in Household Members of Infected HCWs

People sharing the same household as the 29 HCWs tested seropositive for SARS-CoV-2 were also included in the study. Demographic and baseline characteristics of the seven volunteers included are depicted in Table 4. Only two (29%) household members had specific antibodies against SARS-CoV-2. Both were the spouses of HCWs who had typical symptoms and a positive RT-PCR on NPS.

**Table 4:** Demographic and baseline characteristics of household members of infected HCWs.

	**1**	**2**	**3**	**4**	**5**	**6**	**7**
**Infected HCWs characteristics**							
COVID-19 symptoms	yes	yes	no	no	yes	yes	no
SARS-CoV-2 RT-PCR	negative	positive	ND	ND	negative	positive	ND
SARS-CoV-2 seropositivity	yes	yes	yes	yes	yes	yes	yes
**Household member characteristics**							
Relationship with HCW	parent	spouse	spouse	spouse	spouse	spouse	child
Age, years	70	61	47	39	38	28	20
Sex	F	M	M	M	F	F	M
Any comorbidity	yes	yes	no	no	yes	no	no
Diabetes	no	yes	no	no	no	no	no
Hypertension	no	no	no	no	yes	no	no
Cardiovascular disease	no	no	no	no	no	no	no
COPD	no	no	no	no	no	no	no
Asthma	no	yes	no	no	no	no	no
Cancer	no	no	no	no	no	no	no
Treatments: NSAIDs, corticosteroids or immunosuppressive therapy	no	no	no	no	no	no	no
BMI	22.03	31.14	23.51	20.45	31.23	20.18	24.34
Smoking, *n* (%)	no	no	yes	yes	no	no	no
COVID-19 symptoms	no	yes	no	no	no	no	no
SARS-CoV-2 RT-PCR	ND	positive	ND	ND	ND	negative	ND
SARS-CoV-2 seropositivity	no	yes	no	no	no	yes	no

*BMI, body mass index; COPD, chronic obstructive pulmonary disease; HCWs, health care workers; ND, not done; NSAIDs, non-steroidal anti-inflammatory drugs; RT-PCR, real time reverse transcription polymerase chain reaction*.

### Nonspecific Cellular Immune Response and Production of Type I and II Interferon in Health Care Workers and Patients Diagnosed With COVID-19

**Figure 5:**
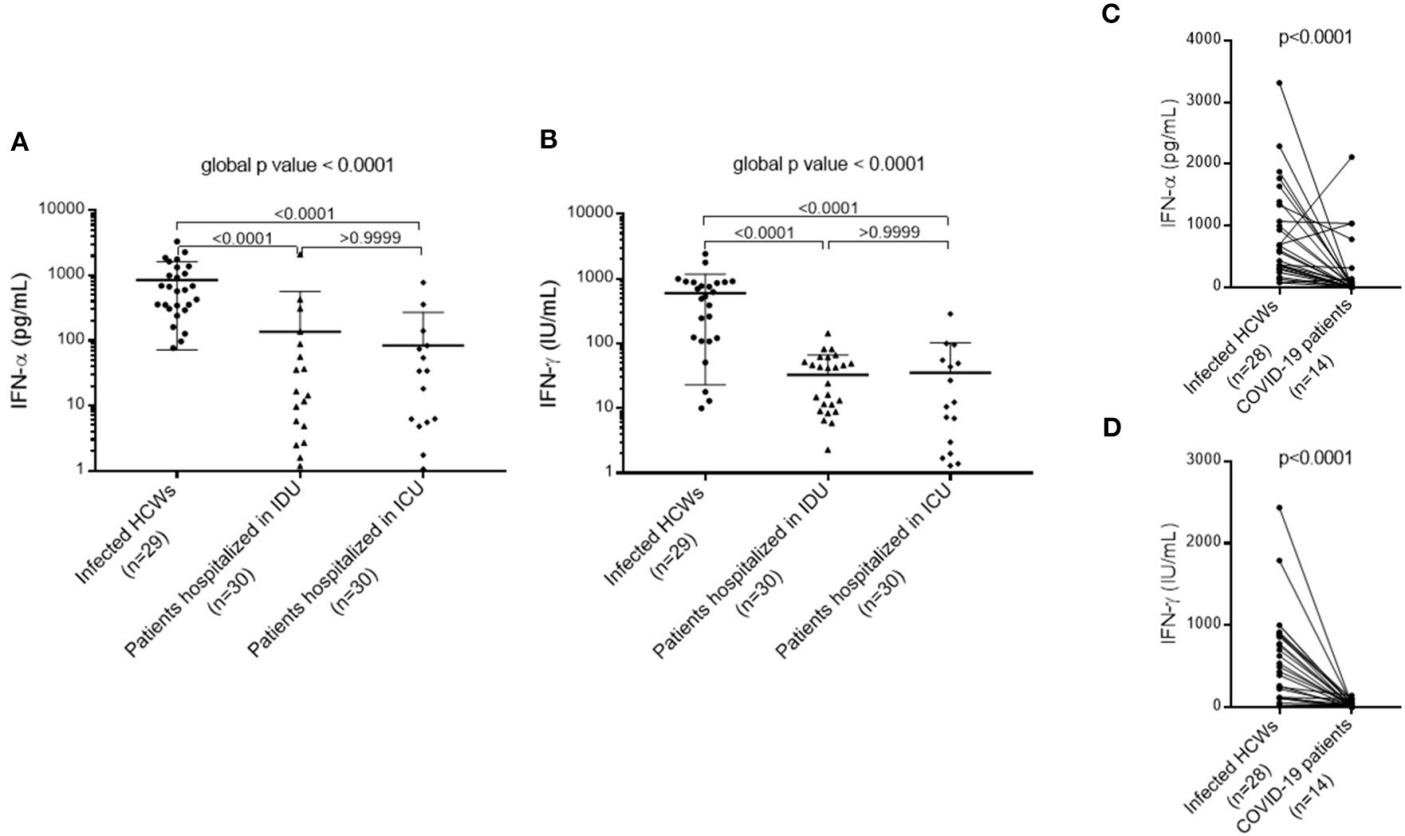
Type I and II interferon response in patients with moderate or severe SARS-CoV-2 infection compared to infected HCWs. **(A)** Type I interferon (IFN-α) response in patients with moderate or severe COVID-19 compared to infected HCWs. **(B)** Type II interferon (IFN-γ) response in patients with moderate or severe COVID-19 compared to infected HCWs. A non-parametric Kruskal-Wallis test was used to compare the three subgroups and obtain a global *p* value. A Dunn's multiple comparisons test was used to compare the subgroups in pairs. **(C)** Type I interferon (IFN-α) response in severe COVID-19 patients after matching (1:2) for age and gender with infected HCWs. **(D)** Type II interferon (IFN-γ) response in severe COVID-19 patients after matching (1:2) on age and gender with infected HCWs. A Wilcoxon matched pairs signed rank test was used to compare IFN-α and IFN-γ levels in infected HCWs to COVID-19 patients. HCWs, health care workers; ICU, Intensive care unit; IDU, Infectious diseases unit.

To evaluate cellular immune responses of pauci- and asymptomatic HCWs, we stimulated whole blood samples from 29 HCWs and 60 patients (with moderate and severe symptoms) diagnosed with COVID-19 with immune ligands and analyzed levels of the cytokines IFN-α and IFN-γ secreted by innate and adaptive cells. When compared to COVID-19 patients with moderate or severe symptoms, innate and adaptive cells of infected HCWs, whether symptomatic or presenting mild symptoms, secreted significantly more IFN-α [infected HCWs: 602.00 (309.00–1335.00) pg/mL; patients in IDU: 7.76 (0.58–51.53) pg/mL; patients in ICU: 6.28 (1.06–74.30) pg/mL, *p* < 0.0001] and IFN-γ [infected HCWs: 537.00 (115.50–886.00) IU/mL; patients in IDU: 16.30 (7.45–50.50) IU/mL; patients in ICU: 7.15 (1.33–48.25) IU/mL, *p* < 0.0001), which suggests impaired type I and II interferon response in patients with moderate or severe SARS-CoV-2 infection (Figures 5A,B). Using ROC-Curve we defined a threshold below 93.00 pg/ml for IFN-α and below 12.10 IU/mL for IFN-γ associated with hospitalization with a sensitivity of 84 and 51%, respectively, and a specificity of 96 and 96%, respectively, (*p* < 0.0001, AUC = 0.93 and *p* < 0.0001, AUC = 0.92, respectively, Supplementary Figure 1). No difference in IFN-γ secretion was found between infected and uninfected HCWs (*p* = 0.4684, data not shown). Because of a higher proportion of women and young subjects among the HCWs compared to the hospitalized patients (Tables 2, 3), we matched the HCWs and hospitalized patients for age and gender using a 2:1 ratio. After matching, we found the same results as before: infected HCWs produced significantly more IFN-α and IFN-γ after nonspecific stimulation than patients with moderate or severe symptoms (Figures 5C,D). Moreover, immune stimulation with CD3 agonist during active infection could induce immune cells apoptosis and explain the IFN defect measured. To verify this hypothesis, we perform a cell count before and after stimulation in 3 patients with COVID-19 (2 severe and 1 moderate form). We did not observe any significant difference in the number of live and dead cells on anti-CD3 agonist stimulated blood compared to unstimulated blood (*p* = 0.1732, Supplementary Figure 2).

## Discussion

SARS-CoV-2 is an emerging virus responsible for the COVID-19 pandemic that has spread rapidly around the world. The clinical features and immune responses, both humoral and cellular, of frontline health care workers infected with SARS-CoV-2 have not yet been well described. To better understand the immune responses of this particularly exposed population, we compared the results to those obtained on a cohort of patients from the same hospital, and therefore from the same geographical location, and after matching on age and sex. As of May 26, 2020, of the 196 HCWs tested, 29 (15%) had specific antibodies against SARS-CoV-2 and 45% of these 29 seropositive HCWs have been strictly asymptomatic. These results are comparable to those obtained in other studies performed on frontline HCWs, at the same time and under the same conditions with IgG serology coupled with IgA and/or IgM serology (15–17). The significant proportion of asymptomatic infected subjects transmitting the SARS-CoV-2 (2–4) and the relatively high seroprevalence of SARS-CoV-2 infections among frontline HCWs (15–17) suggest that the use of screening strategies based on symptoms alone may not be effective in preventing the introduction and spread of SARS-CoV-2 in a hospital setting. However, in our study only the two HCWs who had typical COVID-19 symptoms with a positive RT-PCR on NPS transmitted the virus to their spouses, while five other infected HCWs, with no or negative NPS, did not transmit the SARS-CoV-2 to their household members. However, the small sample size prevented us from drawing statistically significant conclusions. A large study conducted in the United States showed that out of 498 members of confirmed COVID-19 case's households, 57% were infected with SARS-CoV-2 (18). Another study found, after analyzing viral spread among HCWs and residents of a nursing facility, a weak correlation between symptoms and viral shedding (viral titers from respiratory tract), despite difficulty of determining precise dates of symptoms onset, especially if the subjects were pauci-symptomatic or with atypical symptoms (19). These data strengthen current recommendations for expanded screening of HCWs and the universal use of face masks for all, especially in health care.

In our study, all HCWs included worked in units caring for COVID-19 patients, but there was no difference in the rate of SARS-CoV-2 seroprevalence between HCWs directly or indirectly in contact with infected patients. Indeed, although the contamination conditions have not been clearly identified in our cohort of HCWs (close contact with a COVID-19 patient or with another infected HCW during professional activity, or contamination outside the hospital), the seroprevalence of SARS-CoV-2 infection in frontline HCWs is higher than in HCWs from non-COVID units (1.47% on June 25, 2020 in our hospital) and is higher than the estimated seroprevalence in the general population [5.3% on May 11, 2020 in France (20)]. However, the serologies carried out by occupational medicine in our hospital for staff screening only included the determination of IgG and not IgA and IgM, responsible for a probable underestimation of the number of cases.

Knowing the strength and duration of immunity after SARS-CoV-2 infection would allow a better assessment of individual immune protection and aid in decision making on easing restrictions on physical distancing and wearing of a face mask. Several studies characterizing adaptive immune responses to SARS-CoV-2 infection have reported that most convalescents have detectable neutralizing antibodies, which correlate with the number of virus-specific T cells and decrease within 2 months after infection (5, 6, 11, 21). Confirming these previous studies, we have shown a proportion of seroconversion in COVID-19 patients of 100% after 15 days of hospitalization. We then observed a decrease in SARS-CoV-2-specific IgA antibodies titer from the 4th week, although it remained positive. The SARS-CoV-2-specific IgG antibodies titer remained stable during the 7 weeks of follow-up. In comparison, the IgA and IgG peaks of HCWs were lower, which is consistent with their mild symptoms (6). IgA and IgG levels increased during HCWs follow-up, but most did not reach positivity for IgG levels (IgG OD ratio ≥ 1.1), as shown previously (6). During SARS-CoV-2 infection, the IgA response is earlier, stronger, and more persistent than the IgM response (22, 23), but its protective efficacy is still poorly understood, especially when this IgA response is isolated. It is well known that the IgA response is a crucial first-line defense in mucosal tissue, and SARS-CoV-2 infiltrates mainly mucosal tissues. Sterlin et al. also suggested that IgA-mediated mucosal immunity is an essential defense mechanism against SARS-CoV-2 that may reduce the contagion of human secretions and thus reduce viral transmission (24). Thus, some authors have suggested that vaccination against SARS-CoV-2 should trigger IgA responses (25). This explains why we chose to study the prevalence of SARS-CoV-2-specific IgA antibodies rather than IgM antibodies in our cohort. Additional serological surveys of more symptomatic and asymptomatic individuals and longer follow-up are needed to determine the duration of the antibody response. Moreover, the low IgG levels found, or even the absence of IgG, in asymptomatic individuals reinforce the need for a serological survey including a search for IgA antibodies to study the actual infection rate.

Our investigation showed impaired immune cellular responses, illustrated by a type I and II interferons deficiency, in patients with moderate and severe forms of COVID-19 compared to HCWs with mild or asymptomatic forms. It is already well known that immune responses are altered by aging (26), but these results remain significant after matching for age and sex. Our data confirm the results of the study by Hadjadj et al. (10) which suggests that a deficiency of type I interferon in the blood could be a characteristic of severe COVID-19 and could justify therapeutic approaches combining the administration of interferon and anti-inflammatory therapies. However, it is well known that inflammation leads to a secondary deficit of cellular immunity through the suppression of IL-12 expression. As a result, this lack of type I and II interferons could also be secondary to the infection. Other studies showing mutations in type I IFN-related genes (27) or the presence of neutralizing autoantibodies against type I IFN (28) in patients with severe COVID-19 support the hypothesis of a pre-existing immune deficiency predisposing to severe forms of COVID-19 as described in other context (29). Additional studies are needed to clarify this point. If the hypothesis of a pre-existing immune deficiency is confirmed, the deficiency of type I and II interferons revealed after *in vitro* immune stimulation could be a functional blood immune biomarker predicting the severity of the COVID-19. In addition, this immune assay is applicable for routine use.

Our study brings new data but has several limitations. First, difficulties in determining symptoms may have resulted in misclassification of the severity of COVID-19 in some HCWs and patients. In fact, the collection of HCWs' symptoms was done using a self-questionnaire, which can lead to a memorization bias or on the contrary an overestimation of possible symptoms in this particular context of a pandemic. In addition, some patients were probably wrongly classified in the “moderate form” subgroup because they were hospitalized in IDU because of their advanced age, severe comorbidities, or social isolation and not because of the severity of their COVID-19 symptoms. Second, young women represent most health care professionals, a bias that we tried to cushion by performing age and gender matches with patients. Third, this investigation is single-center, carried out only in units caring for COVID-19 patients, resulting in a small sample size. More studies are needed to better understand the immune response of this population continuously exposed to SARS-CoV-2 infection since the start of the pandemic.

A longer clinical and serological follow-up is essential to investigate the efficacy of the protection induced by an isolated IgA response and study the persistence of the antibodies over time. Thus, HCWs included in this study will benefit from extended clinical and serological follow-up.

Defense against SARS-CoV-2 requires both humoral and cellular immune responses. The more detailed study of the immune response in HCWs, highly exposed to SARS-CoV-2 for a prolonged period of time, could provide a better understanding of the alteration of the immune system of patients with a severe form, and thus manage them better. This knowledge could also allow us to adapt the exposition of HCWs according to their immune profile and the treatment in case of infection preventing the evolution to a severe form of COVID-19 combining the administration of interferon and anti-inflammatory therapies.

## Data Availability Statement

The raw data supporting the conclusions of this article will be made available by the authors, without undue reservation.

## Ethics Statement

The studies involving human participants were reviewed and approved by Comité de Protection des Personnes Sud-Ouest et outre-mer 1. The patients/participants provided their written informed consent to participate in this study.

## Author Contributions

BS-P and MC designed the study. MC and VB carried out experiments. MC, VB, KZ, CF, and CR collected data. MC and BS-P analyzed and interpreted the data. MC, BS-P, and VB drafted and revised the manuscript. All authors contributed to the article and approved the submitted version.

## Conflict of Interest

The authors declare that the research was conducted in the absence of any commercial or financial relationships that could be construed as a potential conflict of interest.

## Supplementary Material

Supplementary Figure 1 IFN-α **(A)** and IFN-γ **(B)** ROC-Curve of infected HCWs vs. patients with severe COVID-19.

Supplementary Figure 2 Cell count before and after *in vitro* stimulation by anti-CD3 agonist in three patients with COVID-19 (two severe and one moderate form).
