# Supplementary figures and images for: Humoral and Cellular Response of Frontline Health Care Workers Infected by SARS-CoV-2 in Nice, France: A Prospective Single-Center Cohort Study
Source: Front Med (Lausanne). 2021 Jan 27;7:608804. doi: 10.3389/fmed.2020.608804 (PMC7873459; doi:10.3389/fmed.2020.608804)

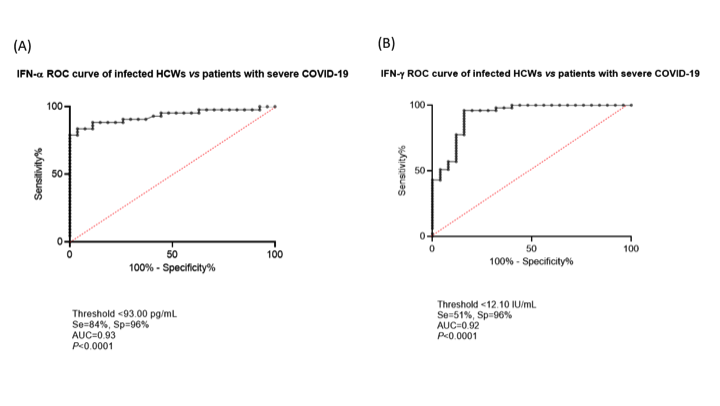

Supplement: Supplementary Figure 1 — IFN-α (A) and IFN-γ (B) ROC-Curve of infected HCWs vs. patients with severe COVID-19. [file Image_1.TIFF]

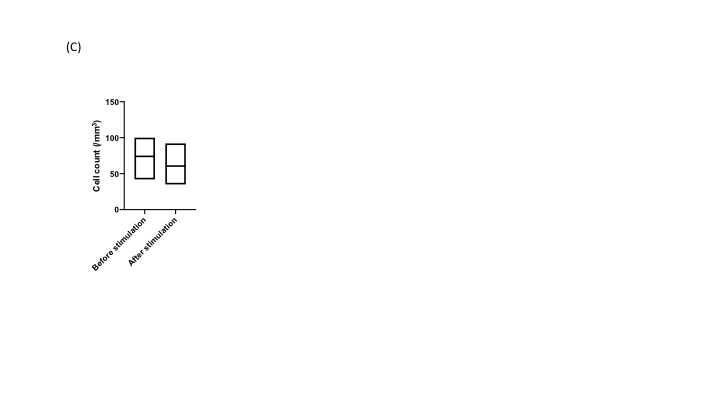

Supplement: Supplementary Figure 2 — Cell count before and after in vitro stimulation by anti-CD3 agonist in three patients with COVID-19 (two severe and one moderate form). [file Image_2.TIFF]
